# CRISPR-Cas9 knockout screening identifies *KIAA1429* as an essential gene in Ewing sarcoma

**DOI:** 10.1186/s13046-023-02828-5

**Published:** 2023-09-28

**Authors:** Kezhe Tan, Wenjie Lu, Feng Chen, Hao Shi, Yingxuan Ma, Zhou Chen, Wei Wu, Zhibao Lv, Jialin Mo

**Affiliations:** 1 Department of General Surgery, Shanghai Children’s Hospital, Shanghai Jiao Tong University School of Medicine, Shanghai, China; 2 Shanghai Key Laboratory of Reproductive Medicine, Department of Histoembryology, Genetics and Developmental Biology, Shanghai Jiao Tong University School of Medicine, Shanghai, China

**Keywords:** Ewing sarcoma, CRISPR-Cas9 screening, *KIAA1429*, *STAT3*, *NKX2-2*

## Abstract

**Background:**

Ewing sarcoma (ES) is an aggressive childhood bone and soft tissue cancer. *KIAA1429* is one type of N6-methyladenosine (m6A) writer that plays a tumor-progressive role in various cancers, but the role of *KIAA1429* in ES remains to be elucidated. The aim of the study was to investigate the role of KIAA1429 in ES.

**Methods:**

We performed a multi-omic screen including CRISPR-Cas9 functional genomic and transcriptomic approaches, and identified that *KIAA1429* played a significant role in ES progression. Gene knockdown, quantitative real-time PCR (Q-RT-PCR), immunoblotting, CellTiter-Glo assays, clonogenic assays, a subcutaneous xenograft model and immunohistochemistry were used to assess the functional role of *KIAA1429* in ES. We mainly conducted RNA sequencing (RNA-seq) in ES cells to analyze the downstream regulatory mechanism of *KIAA1429*. An integrative analysis of chromatin immunoprecipitation sequencing (ChIP-seq) and RNA-seq indicated the upstream regulatory mechanism of *KIAA1429*.

**Results:**

In vitro and in vivo CRISPR-Cas9 knockout screening identified *KIAA1429* as an ES-dependent gene. Genetic suppression of *KIAA1429* inhibited ES cell proliferation and tumorigenicity both in vitro and in vivo. Further studies revealed that *KIAA1429* promotes ES tumorigenesis by regulating the ribosome-associated cell cycle and cancer-related inflammation. Interestingly, we found that *STAT3* was a target of *KIAA1429* and that a *STAT3* inhibitor reduced *KIAA1429* transcript levels, indicating positive feedback between *KIAA1429* and *STAT3*. Finally, we found that *NKX2-2* bound to the *KIAA1429* promoter and transactivated *KIAA1429*.

**Conclusion:**

Our study systematically analyzed ES-dependent epigenetic/transcriptional regulatory genes and identified *KIAA1429* as a biomarker of tumor progression in ES, providing a potential therapeutic target for treating ES.

**Supplementary Information:**

The online version contains supplementary material available at 10.1186/s13046-023-02828-5.

## Introduction

Ewing sarcoma (ES) is generally recognized as an aggressive cancer of bone (approximately 70%) and soft tissue (approximately 30%) that affects children, adolescents and young adults [1, 2]. ES is molecularly characterized by a fusion of an FET gene and an ETS gene, and the most common fusion type is EWS-FLI1 [1, 2]. Despite the fact that therapeutic developments in ES over the past decade have improved the 5-year overall survival rate (~70%), many patients suffer from severe bone ache, and more than 25% of patients are diagnosed with distant metastases, impairing quality of life and finally reducing survival [1, 3]. Most patients with ES usually show relapse within 2 years of initial diagnosis and require complex therapeutic strategies, including chemotherapy, surgery and other therapies [2]. However, these approaches are sometimes limited and are followed by a variety of complications, such as loss of appetite, malnutrition and myelotoxicity in chemotherapy and shock, pain and amputation in surgery [4]. In general, ES is difficult to treat, and it is urgent to further explore the mechanism of ES to find new treatment alternatives.

Epigenetic/transcriptional regulation including DNA methylation, histone modification, nucleosome remodeling, and modulation of three-dimensional chromatin structure, is critical to physiologic and pathophysiologic control of cell fate and proliferation [5, 6]. Multiple previous studies have indicated that epigenetic control plays a pivotal role in ES progression [6]. In particular, previous pan-cancer genome studies have shown high-frequency epigenetic/transcriptional-associated mutations and low-frequency somatic mutations in childhood tumors, indicating that epigenetic/transcriptional-associated pathogenesis and targeted therapeutic strategies are worthy of further study [7, 8]. The N6-methyladenosine (m6A) modification is an epigenetic modification that refers to the addition or removal of a methyl group to/from the nitrogen the 6th carbon of adenine nucleotide, which is the most common epigenetic modification in RNA molecules [9]. The m6A network involves writers such as *METTL3* and *METTL14*, readers such as *IGF2BP3* and *YTHDF2*, and erasers such as *ALKBH5* and *FTO* [10]. Previous studies showed that the m6A process is involved in cancer progression; however, few reports on this topic have been found for ES [11].

Based on CRISPR-Cas9 screening of whole genome and of epigenetic/transcriptional-targeted genes *in vitro* and *in vivo*, our study identified *KIAA1429* (also called Vir Like M6A Methyltransferase Associated, *VIRMA*), a “writer” of m6A modification [9], as an ES-dependent gene. Furthermore, we demonstrated that *KIAA1429* was characterized by high expression and tumorigenic dependence and promoted ES proliferation in *in vitro* and *in vivo* models. We also used transcriptomic and epigenomic approaches to further investigate the upstream and downstream mechanisms underlying *KIAA1429* in ES.

## Materials and methods

### CRISPR-Cas9 knockout screening

CRISPR-Cas9 screening was performed as described previously [12]. Briefly, human ES cells (A673 and SKNMC cells) were transduced with lentiCas9-Blast (Addgene, 52962) (MOI<0.7) to generate Cas9-expressing cells, and then the cells were transduced with an sgRNA library targeting epigenetic/transcriptional regulatory genes (MOI < 0.3). The sgRNA library contains 6,528 sgRNAs (1,103 epigenetic/transcriptional genes with 6,379 sgRNAs and 149 nontargeting control (NTC) sgRNAs)** (**Supplementary Table 1). Transduced cells were subsequently cultured *in vitro* or subcutaneously injected into *Nu/Nu* mice for viability screening *in vitro* or *in vivo*. Genomic DNA was extracted from cultured cells and subcutaneous tumors for sequencing and was further analyzed by the MAGECK algorithm [13] (Supplementary Table 2).

### Cell culture

ES cell lines A673 and SKNMC were purchased from the Cell Bank of Chinese Academy of Sciences. A673 and virus-packaging HEK293T cells were cultured in Dulbecco’s modified Eagle medium/high glucose (DMEM) (BasalMedia, #L110KJ) supplemented with 10% fetal bovine serum (FBS; Sigma, #F2442) and a 1× penicillin streptomycin (P/S) solution (BasalMedia, #S110JV). SKNMC cells were cultured in Eagle's minimum essential medium (MEM) (BasalMedia, #L510KJ) supplemented with 10% FBS and 1×P/S. STR sequencing of ES cell lines was performed by BIOWING Biotech Co., Ltd (Shanghai, China) (Supplemental Table 3).

### Virus packaging and transfection

Lentiviral shRNA plasmids were constructed by inserting target oligonucleotides into the pLKO.1 (Addgene, #10878) plasmid. Plasmid DNA was extracted using a DNA extraction kit (Vazyme, DC112-01). Lentivirus was packaged by transfecting the plasmids with packaging vectors (psPAX and pMD2.G) and PEI MAX solution (Polysciences, #24765) into HEK293T cells. The viral supernatant was then collected, filtered with a 0.45 μm strainer, concentrated with PEG6000 (Sigma, #81253), resolved in PBS and then aliquoted for further transfection. Cells were infected with viruses and selected with puromycin (1 μg/mL, Yeasen, 60210ES25) for 72 hours. The target oligonucleotides are listed in Supplemental Table 4.

### Immunoblotting

Cell samples were lysed in RIPA buffer (Thermo Fisher Scientific, #89900) and protein concentration was quantified using the Pierce BCA kit (Thermo Fisher Scientific, #23225). Denatured proteins (10-20 μg/lane) were separated by sodium dodecyl sulfate polyacrylamide gel electrophoresis (SDS-PAGE) and transferred onto polyvinylidene difluoride (PVDF) membranes. The membranes were then blocked with 5% fat-free milk (BD Biosciences, #232100) in Tris buffered saline containing Tween 20 (TBST), followed by incubation of rabbit anti-human *KIAA1429* (1:500; Proteintech, #25712-1-AP) or rabbit anti-human β-tubulin (1:5000; Abcam, #ab6046) antibody overnight. Then HRP-conjugated goat anti-rabbit IgG (0.2 μg/ml; Pierce, #31460) antibody was used. A luminescence image analyzer (Fujifilm, LAS-4000) was used to visualize the bands after incubation with enhanced chemiluminescence reagents (Millipore, WBKLS0500).

### Quantitative reverse transcription PCR (Q-RT-PCR)

Approximately 2.5x10^5^ cells were lysed with TRIzol reagent (Thermo Fisher Scientific, #TR118) and total RNA was extracted. RNA was reverse transcribed to cDNA with a High-Capacity RNA-to-cDNA kit (Thermo Fisher Scientific, #4387406). Quantitative PCR was performed using an Applied Biosystems QuantStudio™ 5 Real-Time PCR System (Thermo Fisher Scientific, #A34322) with SYBR Green Master buffer (ROX) (Thermo Fisher Scientific, #A25742). GAPDH was used as an internal control, and mRNA levels were calculated using the 2^delta Ct method. The primer sequences are summarized in Supplemental Table 4.

#### Cell viability assay

Cells were seeded in triplicate in 96-well plates at a density of 2000 cells/well in 100 μL of culture medium. The CellTiter-Glo® luminescent cell viability assay (Promega, #G7573) was then performed to assess cell viability on days 0, 2 and 4 according to the manufacturer’s protocol.

#### Colony formation assay

A total of 500 cells/well were seeded in 6-well plates. Fresh medium was added every 5 days in the first 7 days. Half of the medium was removed and then the same amount of new medium was added every 4 days afterward. Generally, colonies were visible after 8-21 days, and the cells were washed with PBS, fixed with a 10% neutralized formaldehyde solution, and then stained with 0.5% crystal violet (Sigma, #C6158-100G) containing 25% methanol.

#### FACS assay

Cell proliferation and apoptosis were measured with Click-iT™ Plus EdU Flow Cytometry Assay Kits (Invitrogen, C10632) and FITC Annexin V Apoptosis Detection Kit (MultiSciences, AT-101) according to the manufacturer’s instructions, respectively. Fluorescence activated cell sorting (FACS) analysis was performed on a CytoFLEX FACS instrument (Beckman Coulter) and the data were analyzed with FlowJo software (FlowJo).

### Tetracycline-inducible VIRMA knockdown (Tet-on)

The sh*KIAA1429* sequences were cloned and inserted into the Tet-on puromycin-resistant plasmid (Addgene,** #**21915). Tet-on-sh*KIAA1429* plasmids were used for virus packaging in HEK293T cells and stably transfected ES cells were established by puromycin selection. *KIAA1429* knockdown was induced *in vitro* using doxycycline (Dox) at a concentration of 200 ng/mL.

### Animal experiments

The Medical Experimental Animal Administrative Committee in Shanghai approved all animal experiments. BALB/c nude female mice (4-6 weeks) were purchased from the Experimental Animal Center of the Chinese Academy of Sciences (Shanghai, China). For subcutaneous cell line xenografts, 2.5 x 10^6^ ES Tet-on-sh*KIAA1429* cells were subcutaneously transplanted in the dorsal flanks of mice on each side. When the tumors reached a volume of approximately 100 mm^3^, the mice were randomly divided into two groups and given water containing 2 mg/mL Dox with 2% sucrose or 2% sucrose as a control. Tumor volume was measured with the formula 1/2 (long axis * short axis^2). Mice with tumors larger than 1,500 mm^3^ were euthanized.

### Histological analysis

Histological analyses, including hematoxylin-eosin (HE) and immunohistochemical (IHC) staining, were performed by Servicebio Biotechnology Company (Shanghai, China). IHC staining was performed using a primary anti-Ki-67 antibody (Servicebio, #GB121499). The stained cells were counted as a percentage of the total positive cells in the five random fields of view using the IHC profiler plugin in ImageJ software (v1.52p, USA).

### Public data acquisition

The transcriptomic datasets of ES tissues (GSE17679, GSE34620, GSE12102 and GSE142162), ES cells (GSE17679 and GSE36133), normal skeletal muscles (GSE17679 and GSE38718) and human mesenchymal stem cells (hMSC) (GSE7888), and chromatin immunoprecipitation sequencing (ChIP-seq) datasets (GSE141493, GSE176400) for Fig. 7D were downloaded from the Gene Expression Omnibus (GEO) database (https://www.ncbi.nlm.nih.gov/geo/). Data on expression and dependency in cancer cell lines were downloaded from the DepMap database (https://depmap.org/portal/; version 22Q2). ChIP-seq datasets in Fig. 6H were downloaded from the Cistrome database (http://cistrome.org/).

### Analysis of RNA sequencing (RNA-seq) and public ChIP-seq data

The analytical approach was previously described [14, 15]. For RNA-seq analysis of our own samples, reads were mapped to the hg38 reference genome using HISAT2. Read counts were generated with HTSeq (version 0.11.1) and fragments per kilobase million (FPKM) values were calculated. ChIP-seq data were mapped and visualized using the Integrative Genome Viewer (IGV; version 2.16.0) software and WashU Epigenome Browse (http://epigenomegateway.wustl.edu/browser/) according to BigWig files on the hg19 genome track.

### ChIP-qPCR analysis

ChIP-qPCR was performed as previously described [16]. Briefly, 1 × 10^7^ cells were prepared for chromatin immunoprecipitation. Subsequently, 10 μg of chromatin was immunoprecipitated with 10 μL STAT3 (Cell Signaling Technology, 12640S) or 1 μL IgG antibody (Cell Signaling Technology, 2729) and 50 μL Pierce™ ChIP-grade Protein A/G magnetic beads. Primers for qPCR were designed according to the peak sequence of STAT3 at the promoter of *KIAA1429* (Fig .6H) via online software (https://bioinfo.ut.ee/primer3-0.4.0/), and the sequences were listed in Supplementary Table 4.

### Identification of differentially expressed genes (DEGs) and construction of a Venn diagram

For public microarray dataset analyses, DEGs were acquired using the Transcriptome Analysis Console (TAC, V4.0.1) software with default settings. For RNA-seq data, DEGs were analyzed using the false discovery rate (FDR) moderated limma test (package “DESeq2” in R). The cutoff was set for DEG selection based on the criterion of |log_2_ (fold change, FC)| > 0.6 with FDR < 0.05 in public datasets or |log_2_FC| > 0.3 with *P* < 0.05 in RNA-seq data. We constructed a Venn diagram using an available online tool (http://bioinformatics.psb.ugent.be/webtools/Venn/) to visualize the overlapping genes.

### Gene set enrichment analysis (GSEA)

GSEA was performed using GSEA 4.1.0 software according to the online instructions (http://www.broadinstitute.org/gsea/index.jsp). All cancer-related gene sets were downloaded from the official GSEA website, which includes gene sets (v7.5) of Hallmark (*n*=50), KEGG (*n*=186), Reactome (*n*=1,654) and Gene Ontology (GO; *n*=10,532). In addition, we extracted the top 500 genes (ranked by *P* value) after FLI1 knockdown in ES cells from GSE61950, GSE94277 and GSE27524, in combination with the top 500 genes upon the dependency score of A673 and SKNMC cells in the DepMap database, and built the gene set “Core ES signature” and other ES-associated gene sets. A NOM *p* value <0.05 or FDR q value < 0.25 was considered statistically significant.

### Protein–protein interaction (PPI) network, Cytoscape and volcano plot

The STRING database (http://string-db.org/) was applied to determine the PPI network of downregulated genes with an interaction value of 0.4, after which Cytoscape combined with the CytoHubba plugin was used to visualize the PPI networks and hub genes. Volcano plots were visualized using ggplot2 package in R.

### Statistical analyses

GraphPad Prism v9.5.1 or R v4.2.1 software was used for statistical analysis. Comparisons between two groups were performed using unpaired two-tailed Student’s *t* test, and comparisons among more than two groups were performed using one-way ANOVA. *In vivo* tumorigenicity was compared between the two groups using two-way ANOVA. *P* < 0.05 was considered to indicate statistical significance.

## Results

### CRISPR-Cas9 knockout screening identifies *KIAA1429* as a critical tumor-dependent gene in ES

**Fig. 1:**
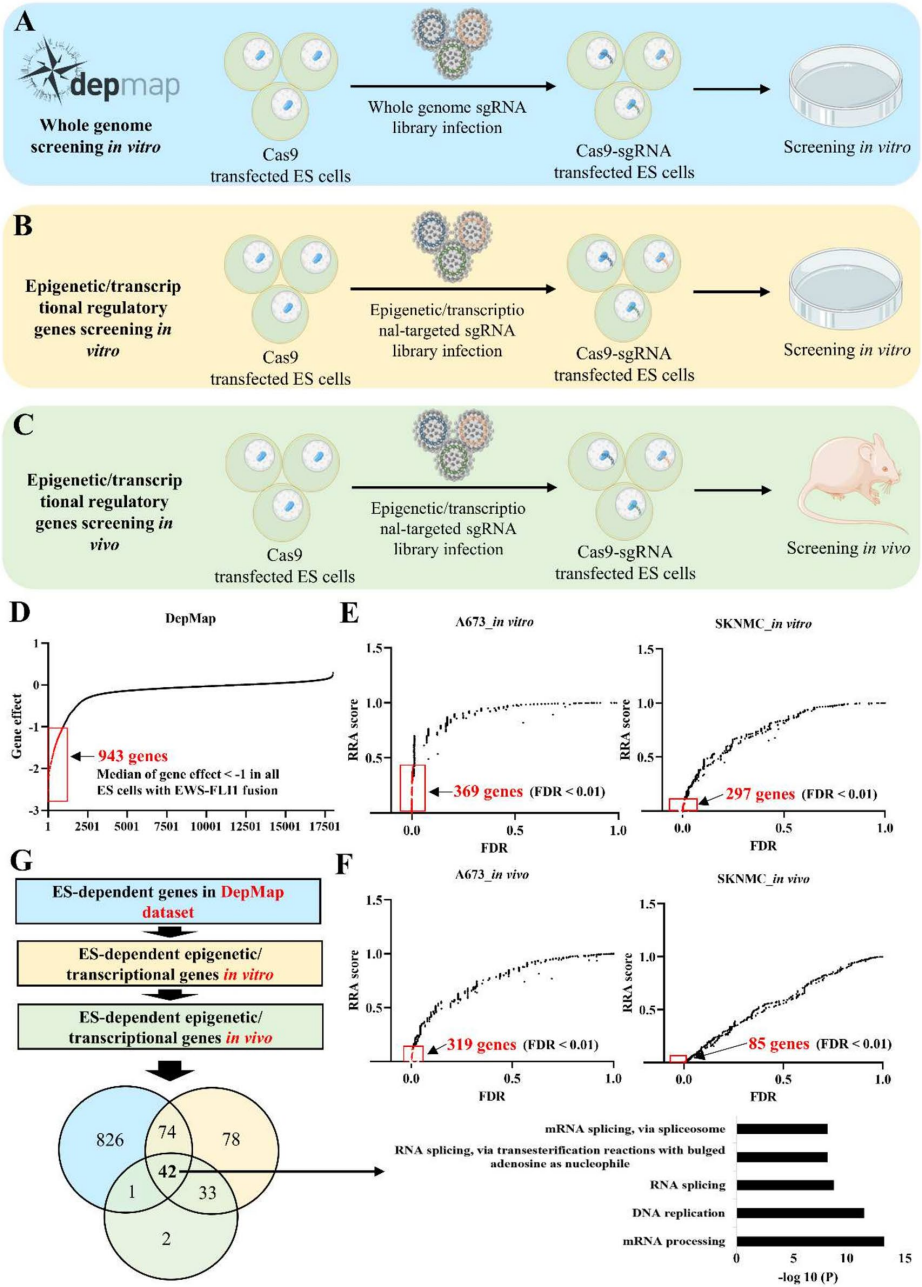
CRISPR-Cas9 knockout screening identifies ES-dependent genes. **A** Schematic diagram illustrating DepMap CRISPR-Cas9 knockout screening in vitro. **B**, **C** Schematic diagram illustrating CRISPR-Cas9 knockout screening targeting epigenetic/transcriptional regulatory genes in vitro (**B**) and in vivo (**C**). **D** Identification of ES-dependent genes in DepMap dataset (13 ES cells with EWS-FLI1 fusion, as shown in Fig. 3A). **E**, **F** Identification of ES-dependent epigenetic/transcriptional regulatory genes in ES cells in vitro (**E**) and vivo (**F**). **G** An integrated analysis of DepMap dataset and CRISPR-Cas9 screen targeting epigenetic/transcriptional regulatory genes. ES: Ewing sarcoma; FDR: false discovery rate

To systematically analyze tumor-dependent genes in ES, we first analyzed the public data of a genome-scale CRISPR knockout screen from DepMap. A total of 943 essential genes were identified in 13 ES cells with *EWS-FLI1* fusion** (**Fig. 1A and D**)**. Previous pan-cancer analysis demonstrated that mutation frequencies in childhood cancers were much lower than those in adult cancers, and genes linked to epigenetic modification emerged as the most common [7, 8], suggesting that epigenetic regulation plays a crucial role in pediatric tumors. Thus, we focused on tumor-dependent epigenetic/transcriptional regulatory factors in ES. We performed CRISPR knockout screening targeting epigenetic/transcriptional regulatory factors in two most commonly used ES cells A673 and SKNMC *in vitro*, and identified 369 and 297 tumor-dependent genes in A673 and SKNMC, respectively **(**Fig. 1B and E). To further narrow down the tumor-dependent genes of ES *in vivo*, we performed *in vivo* CRISPR knockout screening. A total of 319 and 85 tumor-dependent genes were identified in A673 and SKNMC cells, respectively (Fig. 1C and F). Comprehensive analysis of the above conditions identified 42 ES-dependent epigenetic/transcriptional regulatory factors (Fig. 1G, Supplementary Table 5). Among them, several known ES-dependent genes including *AURKB, CDK9, MYC, RPA2, SSRP1, SUPT16H* were found [17–21]. Functional enrichment analysis showed that RNA processing was the most significantly enriched term (Fig. 1G).

**Fig. 2:**
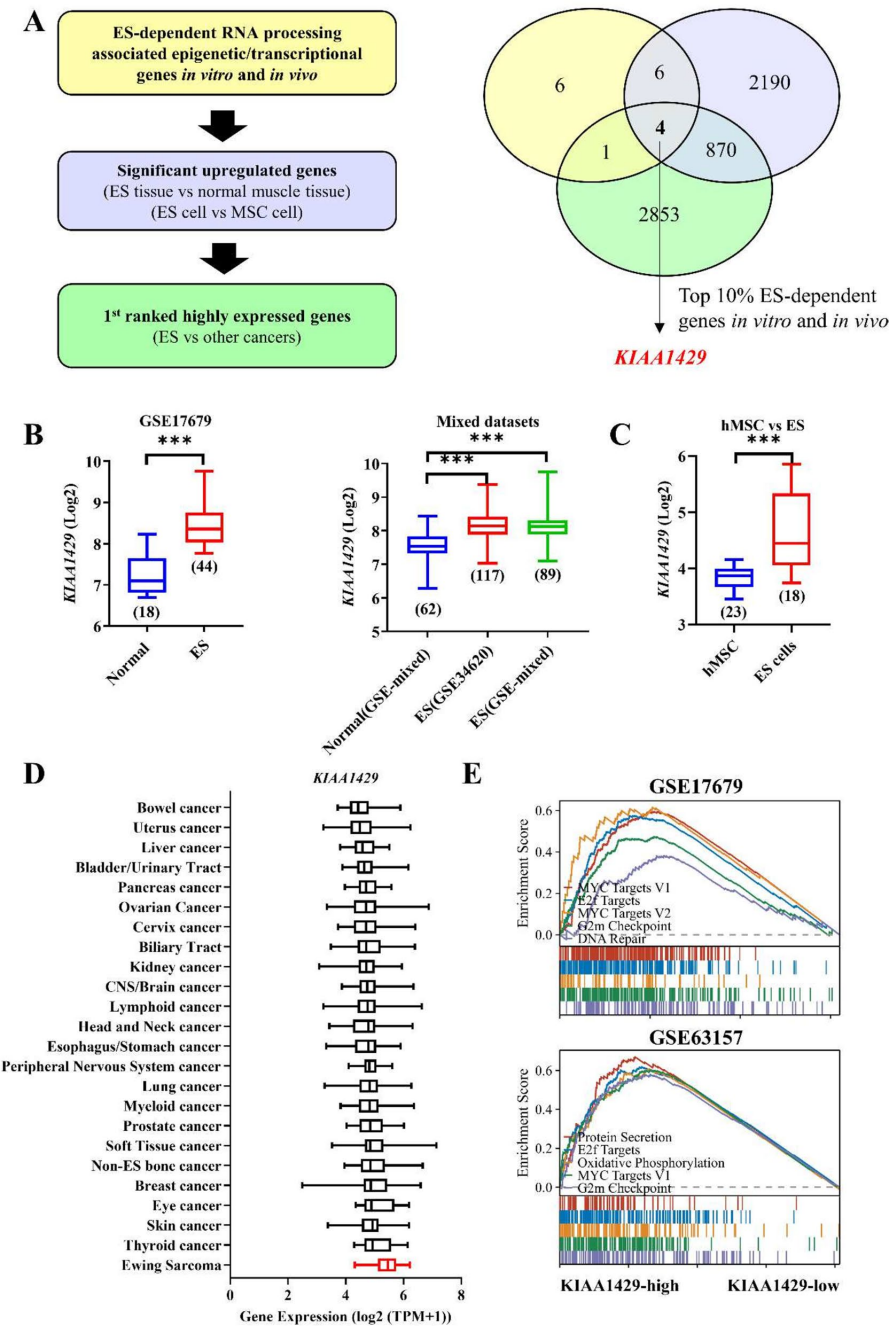
KIAA1429 is identified as an ES-dependent biomarker. **A** Identification of the essential epigenetic/transcriptional regulatory genes in ES. The yellow section indicated ES-dependent RNA processing genes in our sgRNA library. The purple section indicated: (1) significantly upregulated genes in ES tumor tissues than in normal soft tissues in all four tested GEO datasets (log2FC > 0.6, FDR < 0.05 for ES tissue datasets GSE17679, GSE34620, GSE12102 or GSE142162 versus normal soft tissue dataset GSE38718); (2) significantly upregulated genes in 18 ES cell lines than in normal human mesenchymal stem cells (hMSCs) (log2FC > 0.6, FDR < 0.05 for ES cell datasets GSE17679 or GSE36133 versus hMSC dataset GSE7888). The green section indicated 1.^st^ ranked genes in the pan-cancer analysis from CCLE datasets. **B**-**D** Transcriptional profile of ES in tumors (**B**), cells (**C**) and among all cancer cell lines in the DepMap database (**D**). **E** Representative “Hallmark” molecular signatures revealed by GSEA in 2 ES cohorts. hMSCs: human mesenchymal stem cells. *P < 0.05, **P < 0.01 and *** *P* < 0.001

Then, we focused on analyzing a total of 17 ES-dependent RNA processing associated epigenetic/transcriptional genes based on the following criteria: (1) significantly upregulated in ES tumor tissues (GSE17679, GSE34620, GSE12102 and GSE142162) versus normal control tissues (GSE17679 and GSE38718) in GEO datasets (GPL570 platform); (2) significantly upregulated in 18 ES cell lines (GSE17679 and GSE36133) versus normal control human mesenchymal stem cell (hMSC) cells in a GEO dataset (GSE7888; GPL570 platform); and (3) the highest expression in ES among cancers in pan-cancer analysis from the CCLE dataset. Based on the above conditions, 4 candidate genes (*KIAA1429, RBM14, SNRPD1, SNRPF*) were found **(**Fig. 2A). However, *KIAA1429* was the only gene with an effect score in the top 10% for the CRISPR screening of both A673 and SKNMC cells *in vitro* and *in vivo* (Fig. 2A).

In addition, we detected higher *KIAA1429* expression in ES tissues than in normal muscle tissues (Fig. 2B), and *KIAA1429* expression was higher in ES cells than in human MSCs (hMSCs) (Fig. 2C). In the pan-cancer analysis, *KIAA1429* expression was the highest in ES (Fig. 2D). Notably, ES tissue samples showing high expression of *KIAA1429* were enriched in gene sets associated with oncogenic malignancies in the GSEA (Fig. 2E).

### Inhibition of *KIAA1429* suppresses ES cell growth in vitro and in vivo

**Fig. 3:**
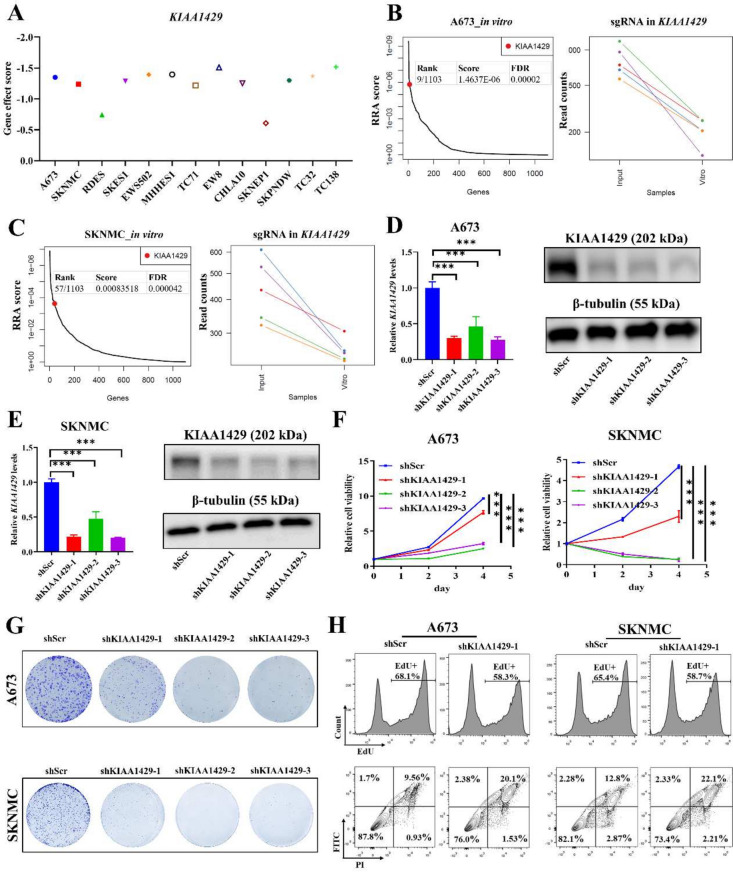
Targeting KIAA1429 suppresses ES growth in vitro. **A** Gene effect data from DepMap showing a strong tumor dependency on KIAA1429 in 13 ES cells with EWS-FLI1 fusion. **B**-**C** Dot plots showing the rank of KIAA1429 (left panel) and parallel coordinate plots showing the change of sgRNA (right panel) in CRISPR-Cas9 screening in vitro in A673 (**B**) and SKNMC (**C**) cells. **D**, **E** KIAA1429 knockdown was measured using Q-RT-PCR and immunoblotting in A673 (**D**) and SKNMC (**E**) cells. **F** The viability of ES cells was measured using CTG after KIAA1429 knockdown. **G** Colony formation of ES cells after KIAA1429 knockdown. **H** Cell proliferation and apoptosis analyses of A673 and SKNMC cells after KIAA1429 knockdown by FACS assay. CTG: Cell-Titer-Glo; ES: Ewing sarcoma; shRNA: short hairpin RNA; shSCR: shRNA scrambled control; sgRNA: small guide RNA. *P < 0.05, **P < 0.01 and *** *P* < 0.001

An analysis of public functional genomics showed that ES tumor growth was highly dependent on *KIAA1429* (Fig. 3A). Furthermore, our CRISPR-Cas9 screening showed the ES tumor viability depended on *KIAA1429* in ES cells (A673 and SKNMC cells) *in vitro* (Fig. 3B, C). We then used shRNA approaches to knock down *KIAA1429* expression in ES cells (Fig. 3D, E), and *KIAA1429* knockdown significantly inhibited ES proliferation *in vitro* (Fig. 3F). Furthermore, colony formation assays showed that inhibition of *KIAA1429* suppressed ES growth (Fig. 3G). We then selected a moderately effective shRNA (sh*KIAA1429*-1) to investigate the effect of *KIAA1429* on cell proliferation and cell apoptosis by FACS staining assays, and the results showed that *KIAA1429* inhibition reduced cell proliferation and induced cell apoptosis in the two ES lines (Fig. 3H).

**Fig. 4:**
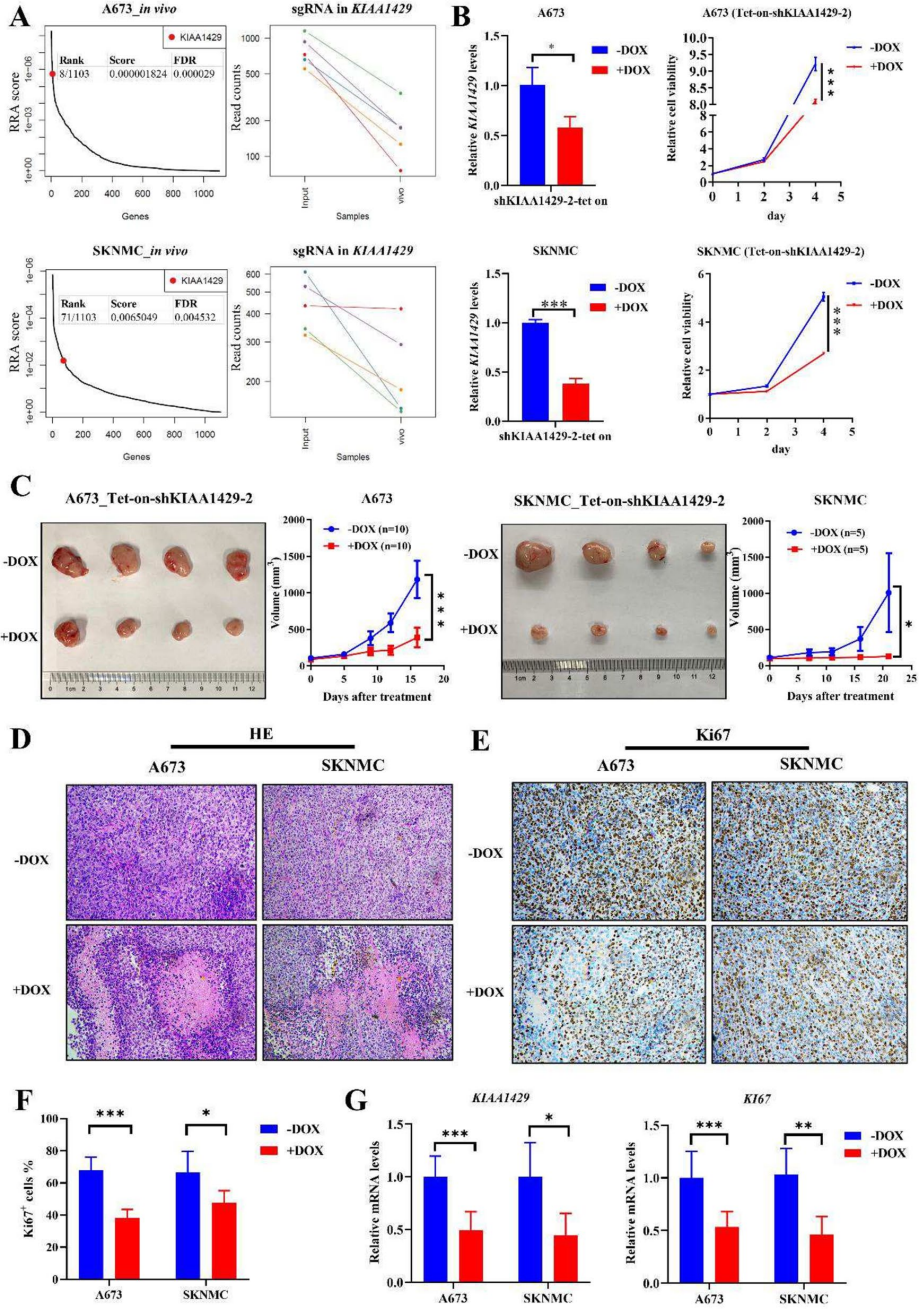
Targeting KIAA1429 suppresses ES growth in vivo. **A** Dot plots showing the rank of KIAA1429 (left panel) and parallel coordinate plots showing the change of sgRNA (right panel) in CRISPR-Cas9 screening in vivo in A673 (upper panel) and SKNMC cells (lower panel). **B** KIAA1429 transcripts and cell viability were measured using Q-RT-PCR and CTG respectively in Tet-on-shKIAA1429 ES cells without or with Dox treatment. **C** Photographs and tumor growth curve of nude mice subcutaneously xenografted with Tet-on-shKIAA1429 ES cells without or with Dox in feed. **D**-**E** Representative images of HE (**D**) and IHC (**E**; Ki-67 staining) in tumor tissue from Tet-on-shKIAA1429 Dox-off and Dox-on mice. **F** Quantification of the percentage of Ki-67 positive cells. **G** KIAA1429 and Ki67 transcripts were measured using Q-RT-PCR in tumors from Dox-off and Dox-on mice. Dox: doxycycline; HE: hematoxylin and eosin; IHC: immunohistochemistry; Tet: tetracycline. * *P* < 0.05, **P < 0.01, and *** *P* < 0.001

Our CRISPR-Cas9 screening showed that ES tumor viability depended on *KIAA1429 in vivo* as well (Fig. 4A). We further validated the inhibitory effect of *KIAA1429* suppression on ES growth *in vivo* by selecting the most powerful shRNA (sh*KIAA1429*-2) to construct a Tet-on system for the induced disruption of *KIAA1429* expression. The results showed that dox-induced *KIAA1429* knockdown suppressed ES proliferation both *in vitro* and *in vivo* (Fig. 4B, C). Histological analysis revealed that *KIAA1429* knockdown disrupted tumor microarchitecture (Fig. 4D) and reduced cell proliferation (Fig. 4F). *KIAA1429* knockdown and inhibited proliferation were validated using Q-RT-PCR assays (Fig. 4G). Overall, inhibition of *KIAA1429* attenuates ES proliferation both *in vitro* and *in vivo.*

### *KIAA1429* promotes ES tumorigenesis through multiple cancer-associated pathways

**Fig. 5:**
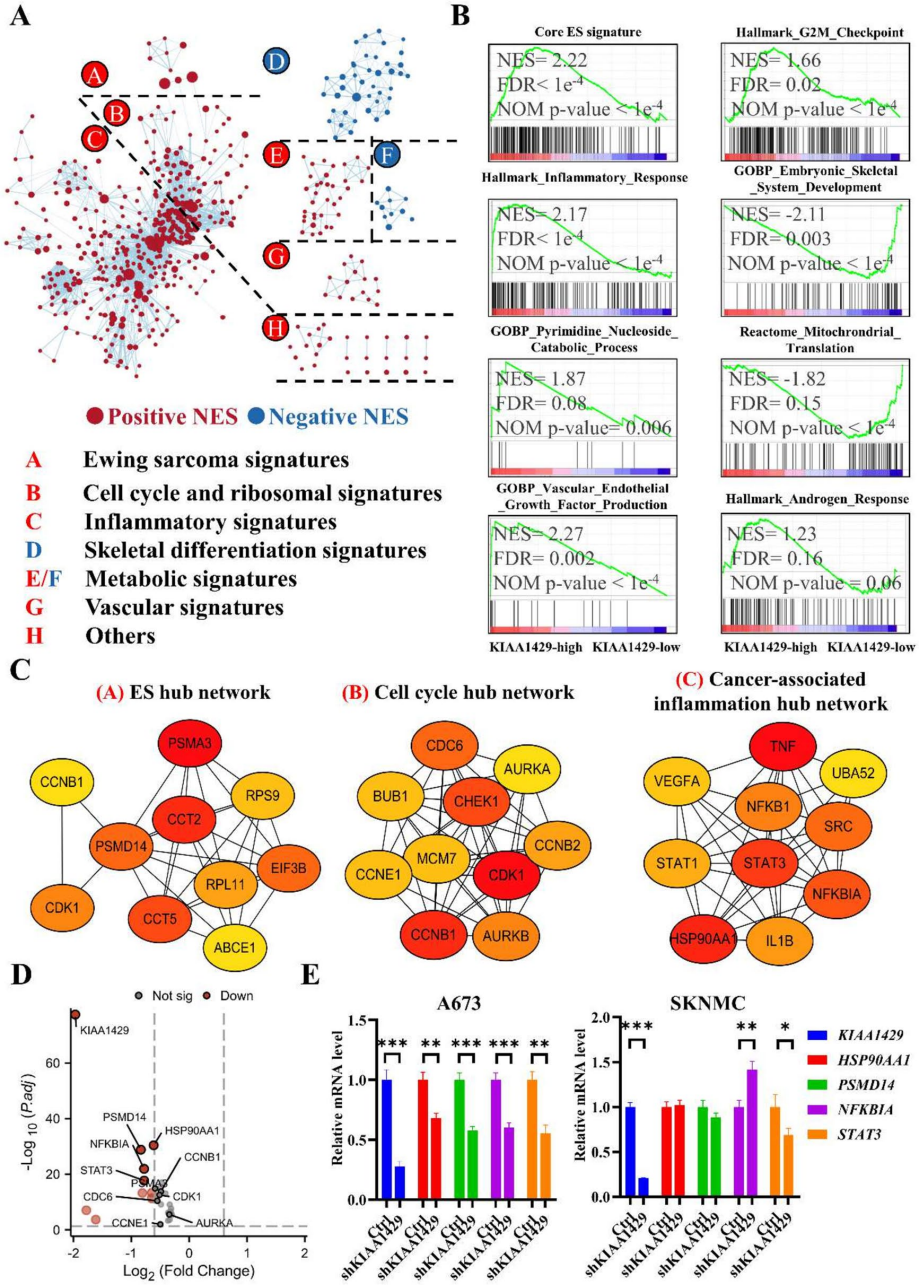
High-expressed KIAA1429 enriches various cancerous pathways in ES. **A** Functional enrichment map depicting the functional groups of the main GSEA hits for the high-expressed KIAA1429 effects on A673 cells based on shKIAA1429 RNA-seq results. **B** Representative GSEA of different populations of gene sets in the KIAA1429-high and KIAA1429-low groups in A673 cells. **C** The “Degree” approach using Cytoscape software (CytoHubba plugin) to show the top 10 hub genes in 3 main populations of gene sets (ES, cell cycle and cancer-associated inflammation). **D** Volcano plots showing the hub DEGs in A673 cells after KIAA1429 knockdown. The cutoff setting was |log_2_FC|> 0.3, *P* < 0.05. **E** Q-RT-PCR validation of some hub genes in ES cells indicating the common decrease of STAT3. DEGs: differentially expressed genes; ES: Ewing sarcoma; FDR: false discovery rate; GOBP: Gene Ontology, biological process; GSEA: gene set enrichment analysis; Q-RT-PCR: quantitative reverse transcription polymerase chain reaction. *P < 0.05, **P < 0.01, and *** *P* < 0.001. Nom *p* value < 0.05 or FDR q value < 0.25 was considered as statistical significance

Next, we performed unbiased transcriptome analyses of ES cells (between shSCR and sh*KIAA1429* samples). GSEA showed that high expression of *KIAA1429* was mainly enriched in terms related to ES, malignant cell cycle, ribosome, inflammation, undifferentiation, metabolism and vascular formation (Fig. 5A, B). The signatures of ES, cell cycle and inflammation were the 3 main downstream pathways in ES cells with high *KIAA1429* expression (Fig. 5A). Therefore, we combined genes extracted from GSEA and *KIAA1429*-downregulated genes, and subjected them to PPI analysis to further identify the top 10 hub genes in gene networks of ES, cell cycle and cancer-related inflammation (Fig. 5C). Furthermore, we ranked the 30 hub genes of the three populations by *P* value and found the great significance of *HSP90AA1, PSMD14, NFKBIA* and *STAT3* (Fig. 5D). We validated the potential downstream genes and found that only *STAT3* expression was suppressed in two *KIAA1429*-knockdown ES cells by Q-RT-PCR (Fig. 5E), suggesting that *STAT3* might play an important role in *KIAA1429*-mediated malignancies.

### *KIAA1429* and *STAT3* might form a positive feedback loop

**Fig. 6:**
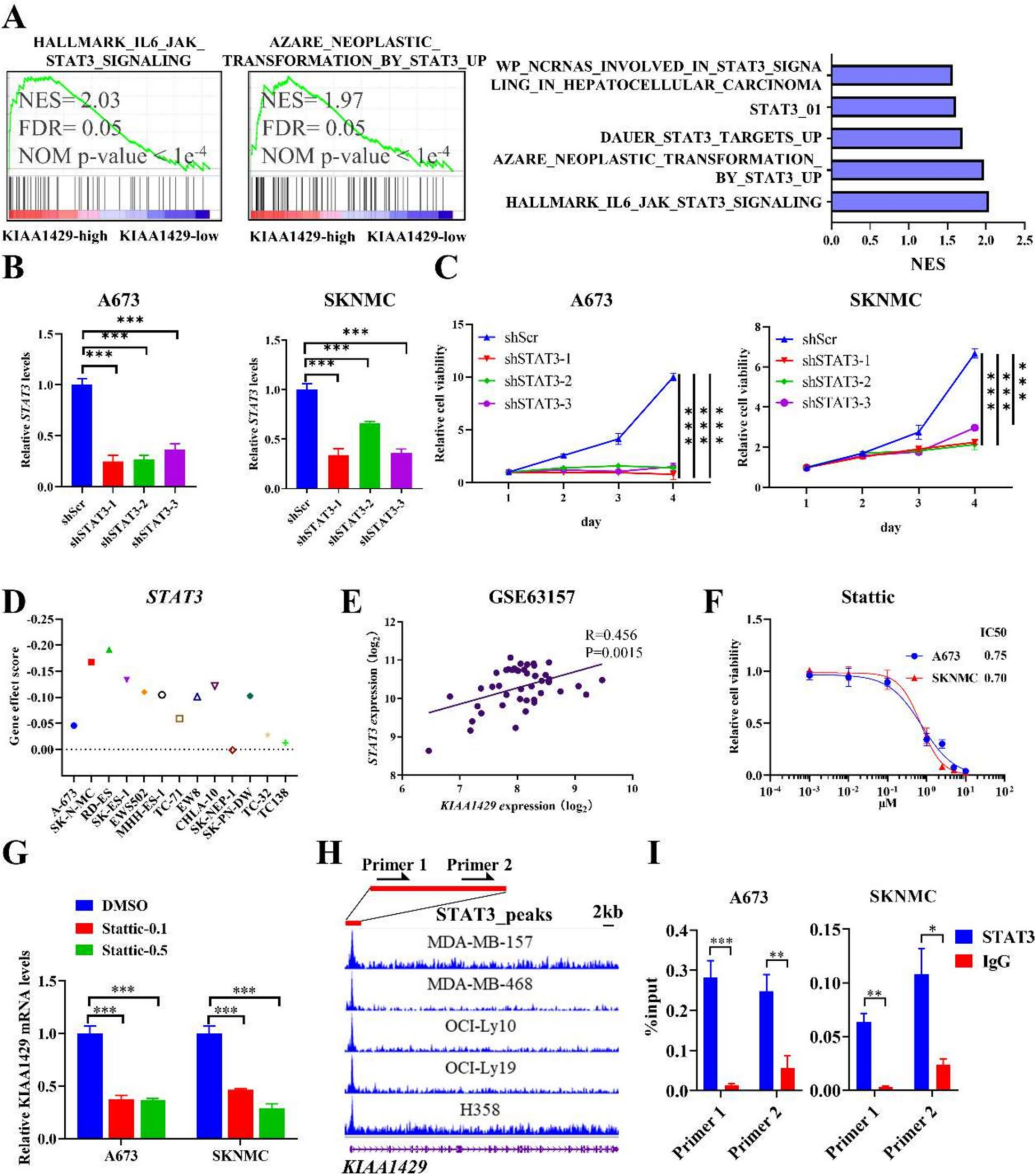
KIAA1429 and STAT3 might form a positive feedback loop. **A** Representative (left panel) and the top 5 (right panel) of GSEA of STAT3-related gene sets in the KIAA1429-high and KIAA1429-low groups in A673 cells. **B** KIAA1429 knockdown in ES cells (A673 and SKNMC cells) was measured using Q-RT-PCR. **C** The viability of ES cells was measured using CTG after STAT3 knockdown. **D** Gene effect data from DepMap showing a relative high level of ES dependency on STAT3. **E** High correlation between KIAA1429 and STAT3 was visualized in one representative ES tumor dataset. **F** High drug sensitivity of STAT3 inhibitor (stattic) in ES cells revealed by killing curve of stattic. **G** KIAA1429 suppression by stattic in ES cells (A673 and SKNMC cells) was measured using Q-RT-PCR. **H** Gene track showing high binding signals for STAT3 at the KIAA1429 promoter region using public tumor ChIP-seq datasets. Breast cancer cells: MDA-MB-157 and MDA-MB-468. Lymphoma: OCI-Ly10 and OCI-Ly19. Lung cancer: H358. Primer 1–2 indicated the primer locations for ChIP-QPCR. **I** ChIP-qPCR analyses of STAT3 signals at the promoter of KIAA1429 in A673 and SKNMC cells. ChIP: chromatin immunoprecipitation; ChIP-seq: ChIP sequencing; ES: Ewing sarcoma; FDR: false discovery rate; shSCR: shRNA scrambled control. *P < 0.05, **P < 0.01, and *** *P* < 0.001

To further analyze the role of *STAT3* in the *KIAA1429*-associated pathway, we performed GSEA concerning *STAT3* signaling and found that high expression of *KIAA1429* enriched the *STAT3*-related signature (Fig. 6A). Knockdown of *STAT3* in ES cells significantly suppressed ES growth (Fig. 6B, C), and public functional genomics data also indicated tumor dependency of *STAT3* in ES cells (Fig. 6D). Additionally, we observed a positive correlation between *STAT3* and *KIAA1429* expression in ES tumors (Fig. 6E). Stattic is a type of *STAT3* inhibitor, and we generated a killing curve by treating ES cells with this agent (Fig. 6F). Interestingly, inhibition of *STAT3* was shown to suppress *KIAA1429* transcript expression (Fig. 6G). Moreover, we analyzed several public ChIP-seq datasets of *STAT3* in multiple cancers (breast cancer, lymphoma, lung cancer), and the results showed conservative binding of *STAT3* at the *KIAA1429* promoter (Fig. 6H), and further ChIP-QPCR experiments validated the aforementioned finding in ES cells (A673 and SKNMC) (Fig. 6I), further indicating a potential positive feedback loop between *KIAA1429* and *STAT3*.

### The *KIAA1429*-related N6-methyladenosine (m6A) network might be induced by *NKX2-2*

**Fig. 7:**
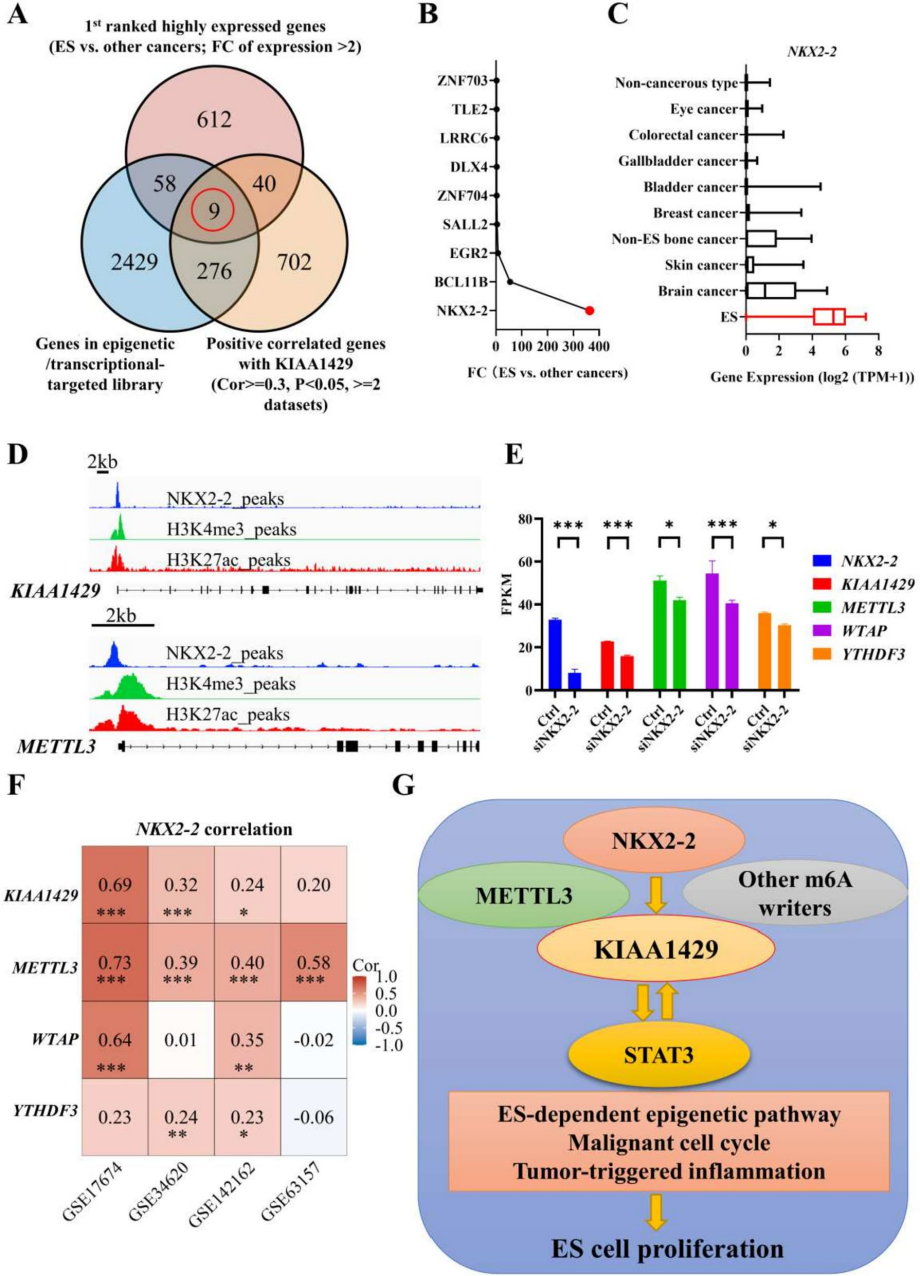
KIAA1429-related m6A network might be induced by NKX2-2. **A** Venn diagram showing the 9 overlapping highly expressed and KIAA1429-correlated transcripts in ES. **B** Ranks of 9 ES-specific transcripts based on the FC in expression. **C** Transcriptional NKX2-2 profile of ES cells among all cancer cell lines in the DepMap database. **D** Gene track showing high binding signals for NKX2-2 at the KIAA1429 promoter region (upper panel) and the METTL3 promoter region (lower panel) using public ES ChIP-seq datasets. H3K27ac and H3K4me3 as the markers of promoter. **E** RNA-seq showing decrease in a set of m6A writers after NKX2-2 knockdown. **F** Matrix showing the gene–gene correlation value among KIAA1429, METTL3, WTAP and YTHDF3 expression in different ES tumor datasets. **H** Summary diagram describing the KIAA1429 pathway and potential critical phenotypes in ES cells. ChIP: chromatin immunoprecipitation; ChIP-seq: ChIP sequencing; ES: Ewing sarcoma; FC: fold change; *P < 0.05, **P < 0.01, and *** *P* < 0.001

Finally, we aimed to explore the epigenetic/transcriptional upstream signaling of *KIAA1429*. A pooled analysis was performed using a DepMap dataset of the 1^st^ ranked highly expressed genes, a gene library of epigenetic/transcriptional regulatory genes [15] and more than two ES tumor datasets of genes positively correlated with *KIAA1429* to identify 9 genes (Fig. 7A). We ranked them by FC in expression (between ES and other cancer types; Fig. 7B), the results suggested that *NKX2-2* might be an upstream regulator in the *KIAA1429*-mediated pathway with high ES specificity (Fig. 7C). Next, we combined one epigenomic dataset and one transcriptomic dataset, and the analysis showed that the m6A writers *KIAA1429* and *METTL3* might be downstream genes regulated by NKX2-2 (Figs. 7D, E). Additionally, a positive correlation between *NKX2-2* and m6A writers was found in different ES tumor datasets (Fig. 7F).

Collectively, the results indicate that *NKX2-2* regulates *KIAA1429*-associated m6A writers, facilitating the positive feedback loop between *KIAA1429* and *STAT3* and reinforcing ES-dependent epigenetic changes, the malignant cell cycle and tumor-triggered inflammation in ES (Fig. 7G).

## Discussion

ES is sensitive to chemotherapy, but the treatment of metastatic and recurrent ES is still a challenge for researchers and clinicians [1–3]. ES is driven by the interplay of epigenetic regulators [6]; therefore, we performed an integrative analysis to identify *KIAA1429* as a potential therapeutic target in ES patients. In addition, we revealed that *KIAA1429* positively regulated ES cell viability and tumorigenicity. In particular, *KIAA1429* regulated various cancer-associated processes and formed a positive feedback loop with *STAT3. NKX2-2* was proved to be an upstream regulator. Taken together, these results indicate that *NKX2-2* triggers *KIAA1429*-associated m6A writers to promote ES progression via the *KIAA1429*-*STAT3* pathway.

*KIAA1429* is believed to be a “writer” of the m6A modification complex that influences RNA modification. Previous studies have shown an oncogenic role for *KIAA1429* in various types of cancers, such as glioma [22], colorectal cancer [23], germ cell tumors [24] and osteosarcoma [25]. To illustrate, Chai et al. [22] concluded that *KIAA1429* was a high-risk gene predicting the prognosis of glioma based on the Chinese Glioma Database. Ma et al. showed that *KIAA1429* enhanced the proliferation of colorectal cancer cells by downregulating *WEE1*, which is that regulated by NK-κB [23]. Miranda et al. demonstrated that *KIAA1429* contributed to germ tumor cell progression and cisplatin resistance [24]. A recent study indicated that *KIAA1429* increased the proliferation, migration and invasion of osteosarcoma cells [25]. In the present study, we also confirmed the tumor-dependent role of *KIAA1429* in ES, further broadening the role of *KIAA1429* in tumorigenesis.

Previous studies have shown that aberrant *STAT3* signaling sustains cell proliferation, facilitates chemotherapeutic resistance, induces metastasis and plays an anti-apoptotic role in ES tumors [26–30]. A completed phase I clinical trial investigated the safety of simvastatin, which was supposed to inhibit *STAT3* signaling (NCT02390843). In addition to ES, different types of *STAT3* inhibitors have been applied in glioma (NCT01904123), breast cancer (NCT03195699), lymphoma (NCT01563302), etc., in clinical trials. In our study, *STAT3* was identified as an oncogenic target gene of *KIAA1429* in ES. Moreover, a transcript-level positive feedback loop between *KIAA1429* and *STAT3* was revealed in the mechanistic study. These results not only reveal the regulatory mechanism of *KIAA1429*, but also provide additional information supporting the theoretical basis for the approval of *STAT3* targeted drugs in clinical trials of ES.

*NKX2-2* has been reported to be a biomarker for ES, and plays critical oncogenic roles in ES progression [31–33]. Notably, *NKX2-2* is a key transcription factor in forming core regulatory circuitry (CRC) to drive important signaling pathways for tumor development [34]. In our study, we further confirmed the highest expression level of *NKX2-2* in ES in the pan-cancer analysis and confirmed nearly undetectable expression in other tumors, as described previously [34]. Epigenome and transcriptome analyses showed that *NKX2-2* directly regulates the transcription of *KIAA1429*. These findings reveal that the restricted expression pattern of *NKX2-2* may induce the specific high expression of *KIAA1429* in ES. Moreover, several m6A-associated functional components, including *MELLT3, WTAP* and *YTHDF3*, were found to be target genes of *NKX2-2*, suggesting that *NKX2-2* probably plays a critical role in the m6A-associated process in ES.

Although epigenetic/transcriptional regulation has been widely reported to be involved in the development, metastasis and drug resistance of various tumors, for most epigenetic/transcription factors, it is difficult to design targeted drugs due to structural disorder and a lack of defined small-molecule binding pockets [35, 36]. However, recent studies have uncovered new targeting strategies for epigenetic/transcription factors; these include direct and indirect approaches, such as the promising direct protein-degradation approach proteolysis targeting chimera (PROTAC) and indirect transcriptional/posttranscriptional modifications [37]. Notably, drug repurposing has risen in popularity, especially in the context of food and drug administration (FDA) approvals and clinical trials, and known pharmacological properties and clinical safety may facilitate the translation of drugs into the clinic [38]. This study provides a novel strategy and direction to identify drugs that specifically inhibit transcription and growth by acting on epigenetic/transcription factors based on a high-throughput drug screening platform. These advanced targeting strategies will provide potential clinical translational significance by revealing new tumor-dependent epigenetic/transcription factors and agents.

## Conclusions

The *KIAA1429*-mediated m6A network facilitates Ewing sarcoma cell proliferation and tumorigenesis via the *STAT3* pathway and is regulated by *NKX2-2*.

### Supplementary Information

**Additional file 1.**

**Additional file 2.**

**Additional file 3.**

**Additional file 4.**

**Additional file 5.**

## Data Availability

The ES tissue transcriptomic datasets: GSE17679 (https://www.ncbi.nlm.nih.gov/geo/query/acc.cgi?acc=GSE17679), GSE34620 (https://www.ncbi.nlm.nih.gov/geo/query/acc.cgi?acc=GSE34620), GSE12102 (https://www.ncbi.nlm.nih.gov/geo/query/acc.cgi?acc=GSE12102), GSE142162 (https://www.ncbi.nlm.nih.gov/geo/query/acc.cgi?acc=GSE142162). ES cell transcriptomic datasets: GSE17679, GSE36133 (https://www.ncbi.nlm.nih.gov/geo/query/acc.cgi?acc=GSE36133). Normal skeletal muscle datasets: GSE17679, GSE38718 (https://www.ncbi.nlm.nih.gov/geo/query/acc.cgi?acc=GSE38718). Human mesenchymal stem cell (hMSC) dataset: GSE7888 (https://www.ncbi.nlm.nih.gov/geo/query/acc.cgi?acc=GSE7888). Chromatin immunoprecipitation sequencing (ChIP-seq) datasets of *NKX2-2*: GSE141493 (https://www.ncbi.nlm.nih.gov/geo/query/acc.cgi?acc=GSE141493). All above datasets were downloaded from the Gene Expression Omnibus (GEO) database (https://www.ncbi.nlm.nih.gov/geo/). Data on expression and dependency in cancer cell lines were downloaded from the Depmap database (https://depmap.org/portal/; version 22Q2). ChIP-seq datasets of *STAT3* were downloaded from the Cistrome database (http://cistrome.org/).

## Abbreviation

BP: Biological process
CC: Cell component
CCLE: Cancer cell line encyclopedia
ChIP: Chromatin immunoprecipitation
COG: Children oncology group
CRC: Core regulatory circuit
CTG: Cell titer Glo
CVS: Crystal violet staining
Dox: Doxycycline
ES: Ewing sarcoma
FC: Fold change
FDA: Food and drug administration
FDR: False discovery rate
GEO: Gene expression omnibus
GO: Gene ontology
GSEA: Gene set enrichment analysis
HE: Hematoxylin and eosin
hMSCs: Human mesenchymal stem cells
IHC: Immunohistochemistry
KEGG: Kyoto encyclopedia of genes and genomes
MF: Molecular function
MOI: Multiplicity of infection
NES: Normalized enrichment score
NOM: Normalized
NTC: Nontargeting control
PPI: Protein to protein interaction
Q-RT-PCR: Quantitative reversed transcription polymerase chain reaction
PROTAC: Proteolysis targeting chimera
shRNA: Short hairpin RNA
shSCR: ShRNA scrambled control
STCs: Stable transfected cells
WT: Wild type
